# A comparison of the recoverable proportion of methicillin-resistant Staphylococcus aureus from two different types of papers

**DOI:** 10.3205/dgkh000266

**Published:** 2016-03-10

**Authors:** Birgul Kacmaz, Serdar Gul

**Affiliations:** 1 Kirikkale University, Medical Faculty, Department of Infectious Diseases and Clinical Microbiology, Yahsihan/Kirikkale, Turkey

**Keywords:** environmental contamination, methicillin-resistant Staphylococcus aureus, MRSA, paper

## Abstract

**Aim:** Paper is used for various purposes in hospitals. Generally, there are two different types of paper, which are commonly used in our facility: wood-free paper, and paper containing wood. We compared the recoverable proportion of methicillin-resistant *Staphylococcus aureus* (MRSA; ATCC 43300) from the surface of such papers.

**Method:** The papers were divided into two groups: Group 1: wood-free paper; Group 2: paper containing wood. The papers were contaminated in a standardized procedure with 0.1 mL of a 5×10^7^ CFU MRSA/mL stock solution.

**Results:** The recoverable proportion of MRSA was higher in the wood-containing papers than in the papers without wood (P=0.043).

**Conclusion:** This study indicates that if paper is purchased for healthcare facilities it should not contain wood, but rather wood-free paper types should be considered.

## Zusammenfassung

**Hintergrund:** Papier wird zu unterschiedlichen Zwecken in Krankenhäusern eingesetzt. Grundsätzlich werden zwei unterschiedliche Arten von Papier in unserer Einrichtung verwendet: Papier ohne Holzanteil und Papier mit Anteilen von Holz. In der vorliegenden Studie haben wir die Rückgewinnungsrate von Methicillin-resistentem *Staphylococcus aureus* (MRSA; ATCC 43300) von der Oberfläche unterschiedlicher Papiere untersucht.

**Methode:** Papier wurde in zwei Gruppen unterteilt: Gruppe 1: Papier ohne Holzanteil; Gruppe 2: Papier mit Holzanteil. Jeweils 1 cm^2^ große Papierstücke wurden in einem standardisieren Vorgehen mit 0.1 mL einer 5×10^7^ KbE MRSA/mL Ausgangslösung kontaminiert.

**Ergebnisse:** Der rückgewinnbare Anteil an MRSA war von Papier mit Holzanteil größer als von Papier ohne Holzanteil (P=0.043).

**Schlussfolgerung:** Die Studie zeigt, dass Papier mit Holzanteil in Gesundheitseinrichtungen nicht verwendet werden sollte.

## Introduction

Health care associated infections remain a major cause of patient morbidity and mortality. Although the most important source of nosocomial pathogens is considered to be the patient’s endogenous flora, recent studies have demonstrated a link between the contamination of patient environments and an increased risk of nosocomial infections [1]. It has been shown that pathogenic bacteria can survive in patient care environments for long periods [1], [2], [3], [4]. Previous studies have shown that pathogenic bacteria may be present on stethoscopes, doctors’ white coats, mobile phones, case notes, patient files, and medical charts [5], [6], [7], [8], [9]. The contamination of patient environments may play a role in the spread of some bacteria, and especially in units where hand hygiene practice is poor. Studies have shown that hand hygiene is the mostly neglected after touching a patient’s surroundings [10], [11]. It is evident that the contamination of environmental surfaces with methicillin-resistant *Staphylococcus aureus* (MRSA) and vancomycin-resistant enterococci (VRE) can be reduced through effective cleaning and disinfection methods. For this reason, not only appropriate hand hygiene is important, but also disinfection of surfaces and items adjacent to the patient. The most frequently touched objects in clinical setting by all health care workers are the patients’ bedside charts, case notes, and patient files. Since those are paper-based materials, it is not possible to clean or disinfect them easily with commonly available liquid disinfectant solutions [12].

Two different types of papers are being used in our hospital: a wood-free paper and a paper containing wood. “Wood-free” is a term used to describe paper that is free from wood particles and lignin. It is also used to describe papers created by chemical pulping. Papers containing wood are created through mechanical pulping or the recycling processes. As the latter processes produce a less refined pulp, a few residual wood particles and lignin remain. Although survival of bacteria on paper was reported previously [12], in this study we investigate the recoverable proportion of MRSA from these two different types of papers.

## Methods

Two different papers were divided into two groups: Group 1: wood-free paper (MOPAK, Karton Sanayi ve Ticaret, Izmir, Turkey); Group 2: paper containing wood (ERKA, Kagit Ticaret, Ankara, Turkey). Samples of 1 cm^2^ (1 cm × 1 cm) were cut from the respective papers, and were steam sterilized. Both study papers were shown to be free of anti-bacterial properties by the manufacturer following DIN 58940-2 [13].

The MRSA strain (ATCC 43300) was cultured overnight and suspended in sterile distilled water. The bacterial concentration was adjusted to 10^8^ colony forming units (CFU)/mL by the photometric measurement of turbidity, which was confirmed by serial dilutions and plating. The final concentration was adjusted to 5×10^7^ CFU/mL. A total of 120 samples (60 samples per study group) were contaminated with 0.1 mL of the stock solution. The samples were stored in a dark, dust-protected climate chamber at 22 ± 2°C and 55 ± 5% relative air humidity. From each group, five contaminated samples were randomly chosen, placed in 10 mL of 0.9% saline solutions, and vortexed. The vortexed solution was diluted 10 times and cultivated in tryptic soy agar (Becton Dickenson, Franklin Lakes, NJ, USA) at 35 ± 2°C for 18–24 hours for colony counts at various time intervals (immediately after drying, day 1, 2, 5, 6, and 7). The number of bacteria per sample was calculated by multiplying the number of counted CFUs with the respective dilution factor. The average of five contaminated papers was taken. The results are given in CFU/cm^2^ paper. Not contaminated papers served as negative control per counting time.

 The statistical analysis was done using SPSS 20.0 (IBM, USA) software package. The differences between the groups were analysed by the Wilcoxon signed-rank test. The significance level of this study was set at p<0.05.

## Results

**Figure 1:**
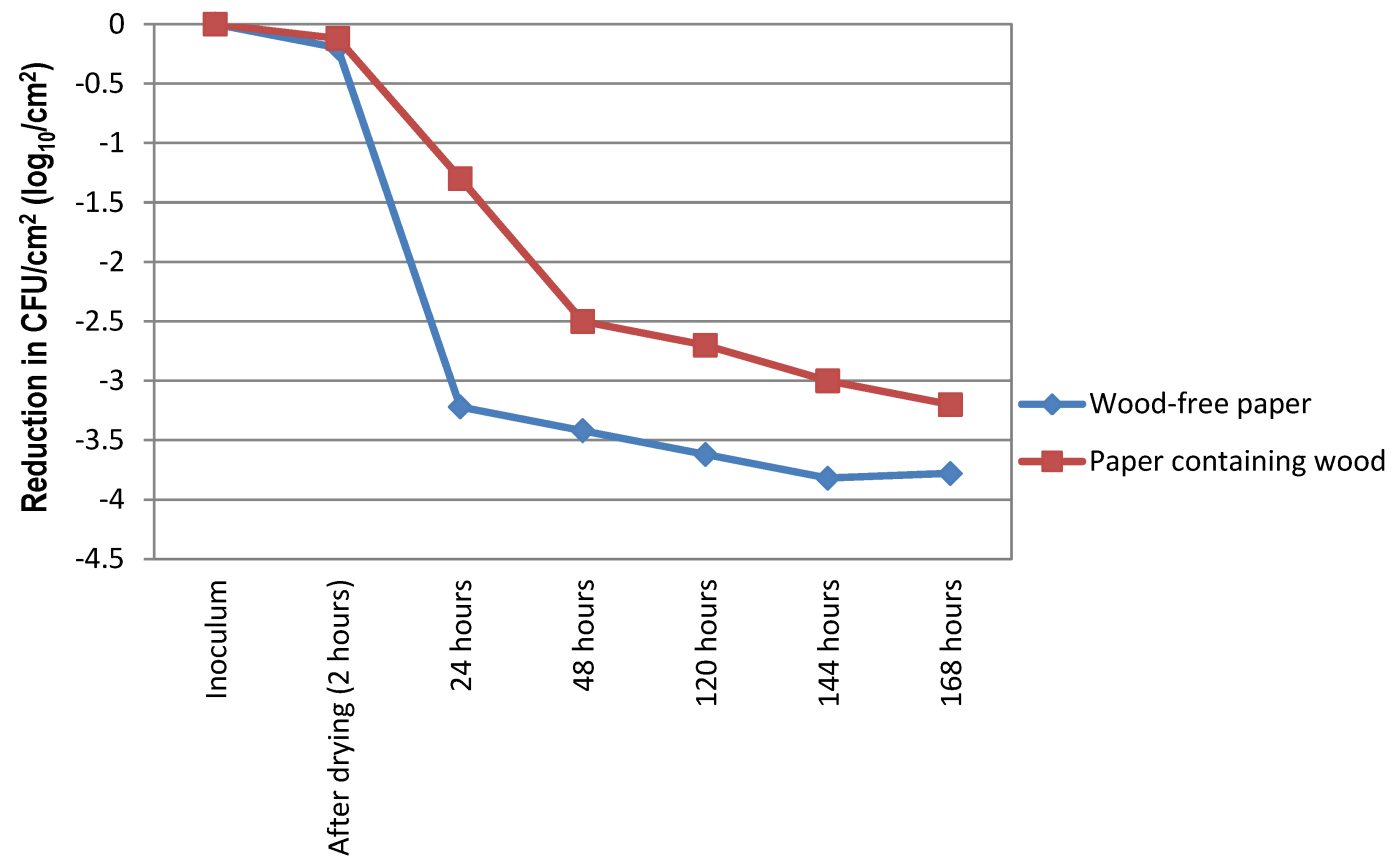
Survival of methicillin-resistant *Staphylococcus aureus* on different types of paper

After drying of the paper, the recoverable proportion of bacteria in wood-free paper (group 1) was 4.1×10^7^ CFU/cm^2^, and in paper containing wood (group 2) 2.3×10^7^ CFU/cm^2^. At the end of this study, in both groups the recoverable proportion of MRSA varied. The recoverable proportion of bacteria in papers containing wood and wood-free papers (CFU/cm^2^ ± standard deviation) were respectively; 1.93±0.41×10^5^, 3.43±0.66×10^4^ at the end of 24 hours (h), 1.25±0.07×10^5^, 11.50±3.53×10^3^ at the end of 48 h, 7.25±1.48×10^4^, 5.00±1.41×10^3^ at the end of 120 h, 2.40±1.55×10^4^, 2.50±2.12×10^3^ at the end of 144 h, 11.00±4.24×10^3^, 2.60±0.56×10^3^ at the end of 168 h. Papers containing wood were showed higher MRSA loads at any investigated time. The recoverable proportion was also higher in this type of papers than in the wood-free papers, and the difference was statistically significant P=0.043 (Figure 1 (Fig. 1)). No growth was determined in the non-contaminated papers with the negative controls.

## Discussion

Paper is widely used in hospitals for various purposes e.g. as a recording medium, for patient files, and reports etc. Paper may be an important vehicle for the cross-contamination of infection in hospital units. Some studies investigating the bacterial contamination in papers highlight that inanimate surfaces including paper may indirectly cause health care associated infections [9], [10], [11], [12]. 

Hübner et al. [12] contaminated white, all-purpose printing papers with pathogenic bacteria and found that these bacteria had survived up to 7 days on the surface of such papers. Furthermore, the authors demonstrated the transmission of bacteria from contaminated hands to paper, and the re-transmission back from paper to the hands in numbers sufficient to cause infection. As a result, the authors concluded that white, all-purpose printing paper may serve as a vehicle for the cross-contamination of pathogens in healthcare settings.

Our study confirms that MRSA may survive during the test period of 7 days on both types of paper. However, we observed that the recoverable proportion of the test strain was higher in the paper containing wood than in the wood-free paper. This difference may be attributable to the varying adhesion capacity of *S. aureus* on the two different surfaces, supported by lignin and wood particles.

Hand hygiene is the most important measure to prevent health care associated infections. However, it is usually poorly performed, and many healthcare worker frequently neglect hand antisepsis after handling medical charts, case notes, and other surfaces made of paper [9]. Since paper cannot be disinfected because of its vulnerability to liquid disinfectants, we believe that the type of used paper may be an important, yet often overlooked aspect.

Our study has some limitations. The investigated papers were contaminated with a methicillin-resistant *S. aureus* strain only. Therefore, we cannot generalise our results to other bacteria, in particular to Gram-negative organisms. Second, although a total of 60 paper samples were contaminated per paper group, only 5 randomly selected paper samples were further processed. However, it is unlikely that a larger sample size may have yielded different results. Furthermore, the transmission of the bacteria from the paper onto hands was not evaluated. It may be that microorganisms harbour better on paper containing wood, but at the same time it may be that such bacteria will be picked up by hands only in smaller numbers compared to paper without wood particle. Although the possibility of re-transmission of bacteria from paper to hands was investigated by other researches in the past [12], we cannot assess the possible impact of the type of paper on re-contamination.

## Conclusion

In conclusion, based on the results of our study, the recoverable proportion was different in the two different types of paper. Since this proportion was higher in paper containing wood, we propose using such material to a lesser degree and for shorter periods in hospitals, especially where hand hygiene practices are poor. If paper is purchased for healthcare facilities it should not contain wood, but rather wood-free paper types should be considered.

## Notes

### Competing interests

The authors declare that they have no competing interests.
